# An in vitro study comparing limited to full cementation of polyethylene glenoid components

**DOI:** 10.1186/s13018-015-0268-7

**Published:** 2015-09-17

**Authors:** R. Andrew Glennie, Joshua W. Giles, James A. Johnson, George S. Athwal, Kenneth J. Faber

**Affiliations:** Department of Orthopedics, Dalhousie University, Halifax, NS Canada; Division of Orthopedics, Western University, 268 Grosvenor St, London, N6A 4L6 ON Canada

## Abstract

**Background:**

Glenoid component survival is critical to good long-term outcomes in total shoulder arthroplasty. Optimizing the fixation environment is paramount. The purpose of this study was to compare two glenoid cementing techniques for fixation in total shoulder arthroplasty.

**Methods:**

Sixteen cadaveric specimens were randomized to receive peg-only cementation (CPEG) or full back-side cementation (CBACK). Physiological cyclic loading was performed and implant displacement was recorded using an optical tracking system. The cement mantle was examined with micro-computed tomography before and after cyclic loading.

**Results:**

Significantly greater implant displacement away from the inferior portion of the glenoid was observed in the peg cementation group when compared to the fully cemented group during the physiological loading. The displacement was greatest at the beginning of the loading protocol and persisted at a diminished rate during the remainder of the loading protocol. Micro-CT scanning demonstrated that the cement mantle remained intact in both groups and that three specimens in the CBACK group demonstrated microfracturing in one area only.

**Discussion:**

Displacement of the CPEG implants away from the inferior subchondral bone may represent a suboptimal condition for long-term implant survival. Cement around the back of the implant is suggested to improve initial stability of all polyethylene glenoid implants.

**Clinical relevance:**

Full cementation provides greater implant stability when compared to limited cementation techniques for insertion of glenoid implants. Loading characteristics are more favorable when cement is placed along the entire back of the implant contacting the subchondral bone.

## Introduction

Glenoid component loosening is a common cause of failed total shoulder arthroplasty (TSA) [1, 2]. Multiple studies have identified factors associated with glenoid component failure including glenohumeral mismatch, glenohumeral instability, excessive glenoid reaming at the time of surgery, cementing techniques, malalignment of the glenoid component, and osteopenic host bone [1, 3].

Although different methods of glenoid fixation are available, clinical and biomechanical studies would suggest that all polyethylene-cemented implants may have better initial in vitro stability and superior mid- and long-term clinical survivorship when compared to metal-backed implants [4–7]. Polyethylene glenoid prostheses can be broadly categorized as either “keeled” or “pegged.” Currently, the cement mantle required for adequate initial fixation and durable long-term survivorship of polyethylene prostheses is not well established [8–12].

Little is known about the effect of various glenoid cementation techniques in total shoulder arthroplasty. Several recent publications examining the effect of pressurization found improved cement interdigitation within cancellous bone that theoretically creates a stronger initial bond to the host bone that may enhance implant stability, minimize radiolucent lines, and increase implant survivorship [13–15]. In addition, Neer suggested that building up cement along the back of the implant lead to poorer implant survival since there was higher potential edge loading and therefore more opportunity for cement fracturing and third body debris in the joint potentially starting the cascade of osteolysis [16]. Others have observed higher implant failure rates when the cancellous bone is exposed for cementation and suggested that preservation of the subchondral plate is critical for implant survival [17]. When the subchondral plate is preserved, there is little opportunity for cement interdigitation with cancellous bone. The purpose of this study was to compare the micro-computed tomography (micro-CT) findings and biomechanical characteristics of two cementation techniques employed during subchondral plate-sparing glenoid preparation. The null hypothesis is that both cementation techniques will demonstrate no significant difference in cement mantle changes on micro-CT and similar biomechanical properties regardless of cementation technique.

## Materials and methods

### Specimen preparation

Sixteen unmatched cadaveric human shoulder specimens were tested (ages 42–75). Each specimen was imaged with radiographs to ensure there were no osseous abnormalities that would prevent component implantation. Seven scapulae were randomized to receive a traditional fully cemented technique with cement around the pegs and the back-side of the implant (CBACK) and nine were randomized to a limited cementing technique only around the implanted pegs (CPEG). Randomization was carried out with a random number generator.

After each specimen was thawed and stripped of soft tissues, the glenoid was prepared to accept a 46-mm pegged prosthesis using the surgical technique provided by the implant manufacturer (Anatomical™, Zimmer, Warsaw, IN). Reaming to create a conforming surface for the implants was performed in a manner that preserved the deep cortical plate in all specimens**.** All scapulae included in the study were size-matched to accommodate a 46-mm implant. The humeral head was simulated using an instrumented steel ball that corresponded to the manufacturer’s recommended radius of curvature mismatch. Third-generation cementation technique was used as described by Reiss and Nyfeller [18, 19]. For the CBACK specimens, cement was injected (Simplex, Stryker, NJ) into the glenoid peg holes and onto the subchondral glenoid bone. Additional cement was intentionally placed on the convex back surface of the component. The cement was then pressurized and the implant inserted. The limited cementation technique (CPEG) injected and pressurized cement into the glenoid peg holes with a syringe. No cement was applied to the convex back surface of the implant or to the glenoid face. Any excess cement that leaked from the peg holes was removed from the back of the implant. In both techniques, the excess cement was removed beyond the margins of the polyethylene and the component was pressed against the glenoid face with an impaction device until the cement was fully cured.

### Mechanical testing of micro-stability

**Fig. 1:**
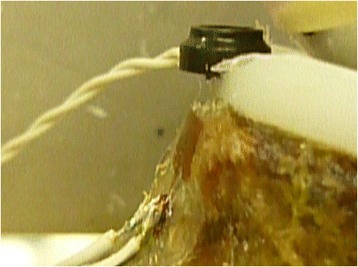
The optical tracker demonstrated on the inferior aspect of the glenoid polyethylene

Glenoid component deformation and differential movement between the component and the adjacent bone was measured using an optical tracking system (OptoTrak Certus, NDI, Waterloo, ON). Two trackers were necessary: a reference tracker was placed on the glenoid bone remote from the implant and the second tracker was placed on the inferior edge of the polyethylene implant. A reference marker was placed on the bone adjacent to the bone-implant interface in order to compensate for all movement of the underlying bone that would otherwise appear as component displacement when recorded by the implant marker (Fig. 1). The optical tracking system was calibrated and confirmed to have a resolution of 0.01 mm and an accuracy of 0.1 mm prior to initiation of testing.

**Fig. 2:**
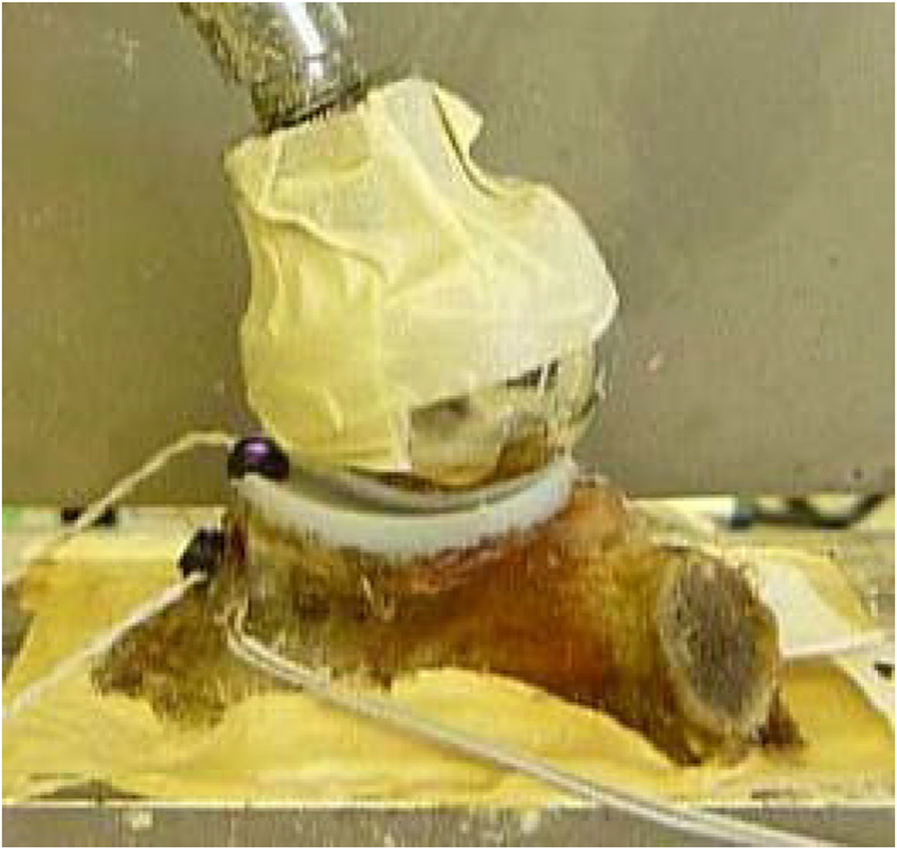
The loading apparatus demonstrates a 30° loading vector with two optical trackers. One optical tracker is attached to the polyethylene and one is attached to the bone as a reference. The scapula is potted within the cement box. There is masking tape over the humeral ball to reduce potential reflection to the camera (not shown)

A sinusoidal cyclic loading protocol was used to continuously load the construct with a 30 degree force vector in the superior direction for a total of 10,000 repetitions at 1250 N. This testing regimen was chosen to simulate 5 high load activities (e.g., rising from a chair, walking with a walker, turning a locked steering wheel, etc.) per day over a 6-year period [20]. Similar loading regimens have been suggested previously [21]. The force vector was achieved using a pneumatic loading apparatus and applied to the glenoid via a custom steel ball with a radius of curvature equivalent to the implant manufacturer’s recommended corresponding humeral head implant (Fig. 2).

Loading and optical tracking data were continuously recorded using LabVIEW software (National Instruments, Austin TX). Mean data at the 50th, 100th, 200th, 500th, 1000th, 5000th, and 10,000th cycle for each group was compared using analysis of variance (ANOVA) in SPSS (IBM, Armonk, NY).

### CT-based radiological assessments

**Fig. 3:**
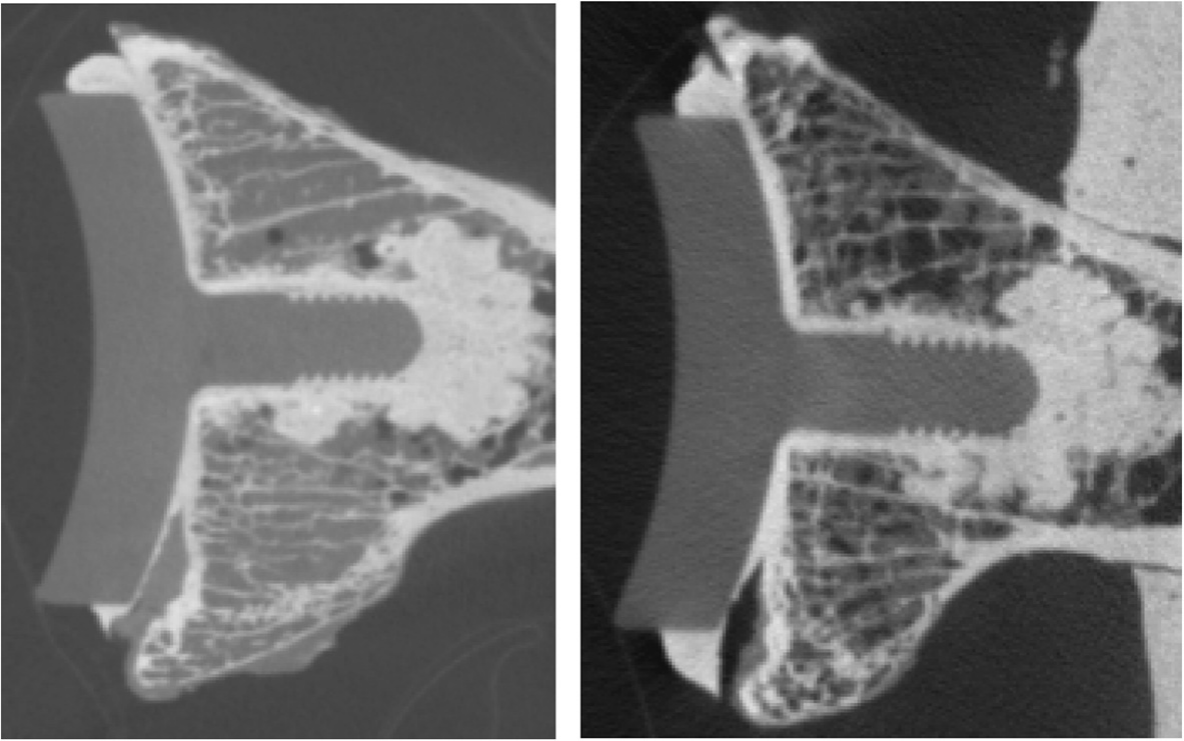
Specimen 8 demonstrates slight change at the anterior portion of the cement mantle interface specifically comparing pre-loading and post-loading CT images

After specimen preparation and before loading, baseline micro-computed tomography (micro-CT) scans were obtained to evaluate the initial incorporation of cement into the glenoid bone surface and in the peg holes. The glenoid samples were imaged using the Locus Ultra micro-CT scanner (General Electric, Fairfield, CT). The scanner has an in-plane field of view of 140 mm in diameter and an axial field of view of 96 mm in length. The samples were imaged with an x-ray source voltage of 120 kV and a current of 20 mA. In a scan time of less than a minute, 1000 views were acquired. The data were reconstructed into a three-dimensional (3-D) image volume with an isotropic voxel size of 154 μm. After completion of the complete loading protocol, the micro-CT scanning was repeated. General Electric Health Care MicroView™ (General Electric, Fairfield, CT) software was used to quantitatively evaluate three-dimensional images of the construct (Fig. 3).

**Fig. 4:**
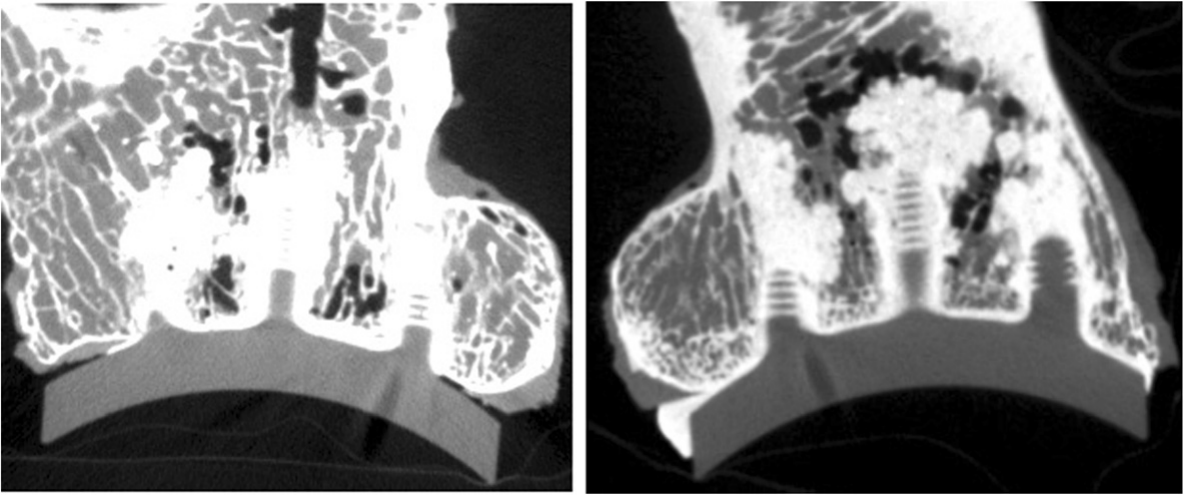
Examples of CPEG micro-CT scan on the *left image* and CBACK on the *image to the right*. The CPEG implant shows no cement along the back of the component whereas the CBACK component shows cement extruding along the undersurface and side of the implant

**Fig. 5:**
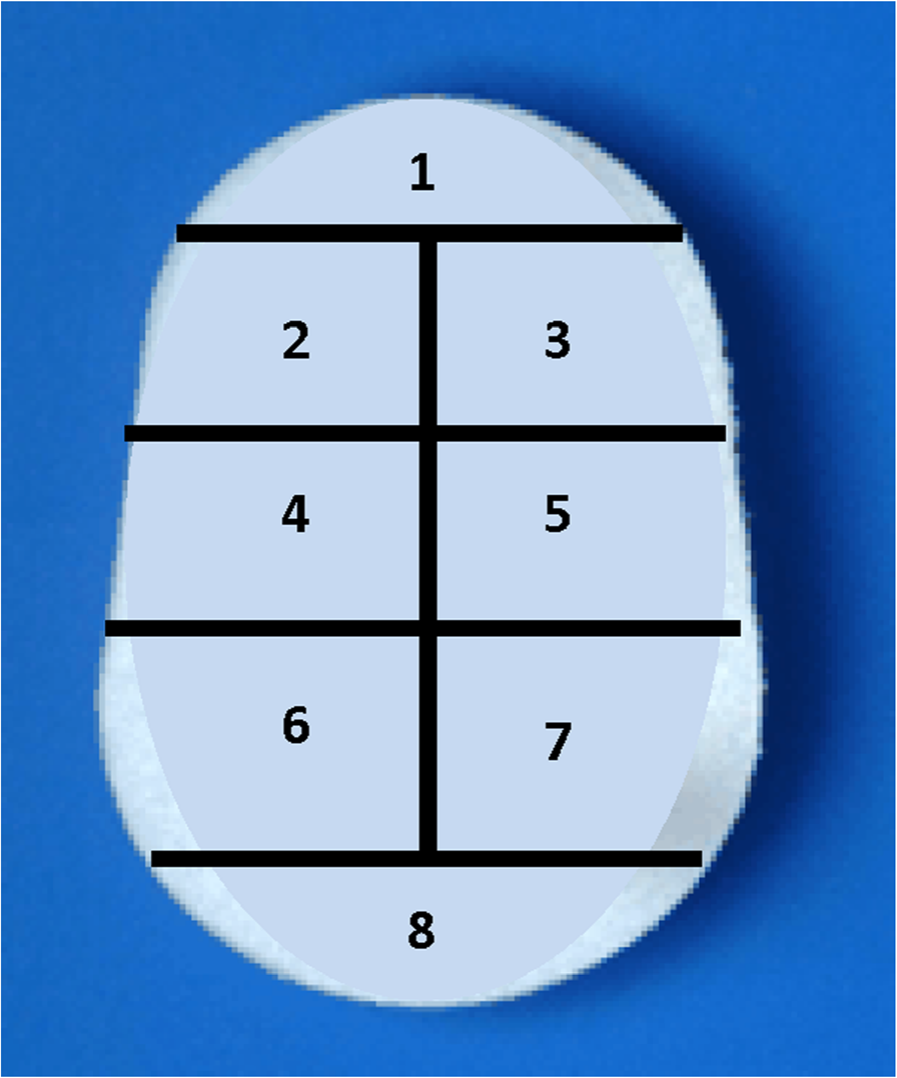
Glenoids were divided into eight zones of interest. The superior peg lies in between zone 2 and 3. The central peg lies in between zones 4 and 5 and the inferior pegs lie in zones 6 and 7

Micro-CT images were evaluated in a random and blinded order and data was recorded using a modified scoring system that was based on the scoring system previously described by Walch [22]. An example of each technique, both CBACK and CPEG, can be found in Fig. 4. Average thickness of the cement mantle was recorded for the CBACK components. Each component was divided into eight different zones that corresponded to positions on the medial surface of the glenoid prosthesis (Fig. 5). A score of 0 was assigned if no radiolucent lines were present within a zone and a score of 1 was assigned if radiolucent lines were present within the zone. A radiolucent line was defined as a visible radiolucency ≥1 mm comparing identical CT images pre- and post-loading. The eight zones of the pre- and post-loading images were compared using chi-squared analysis to determine whether any significant radiolucent lines or cement fractures had developed. All eight zones were carefully scrutinized in each specimen for any evidence of microfracture.

## Results

### Micro-stability testing

One of the specimens from the CBACK group was excluded due to inadvertent camera movement near the beginning of the loading cycle. Therefore, the camera could not visualize the tracker and the data was not recorded.

**Fig. 6:**
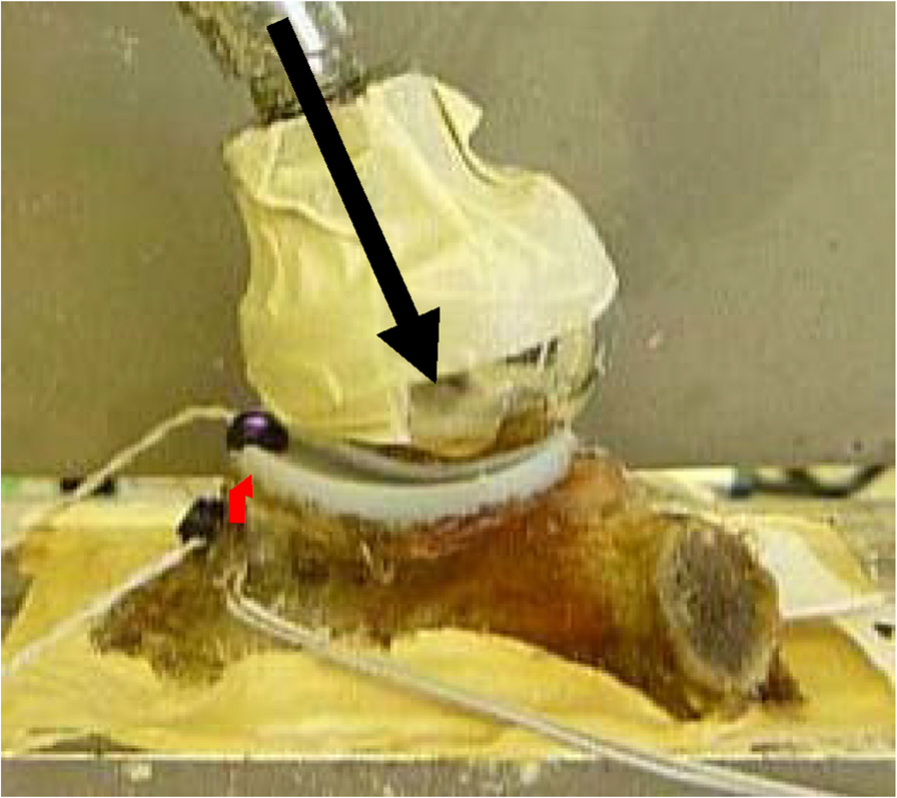
Representation of the glenoid being loaded in a superior direction and demonstrating lift-off as detected by the optical trackers at the inferior portion of the subchondral bone

There was a significant difference in the displacement of the polyethylene implant when comparing CBACK and CPEG cementation techniques dynamically (*p* = 0.03). Physiological loading displaced the implant away from the inferior portion of the glenoid (Fig. 6). The initial mean displacement of the CPEG components at 50 cycles was 0.156 ± 0.038 mm whereas mean displacement of CBACK components was 0.055 ± 0.010 mm (*p* = 0.017). At 10,000 cycles, the mean displacement of the CPEG components increased to 0.255 ± 0.039 mm (*p* = 0.001). This data is summarized in Table 1.

**Table 1:** Mean displacement measurements at different cyclic loading points for both CPEG and CBACK implantation techniques

	CPEG	CBACK
50	0.156 ± 0.114	0.055 ± 0.026
100	0.203 ± 0.094	0.050 ± 0.029
200	0.211 ± 0.097	0.048 ± 0.033
500	0.220 ± 0.097	0.054 ± 0.029
1000	0.233 ± 0.106	0.051 ± 0.035
5000	0.246 ± 0.108	0.052 ± 0.035
10000	0.255 ± 0.118	0.054 ± 0.039

**Fig. 7:**
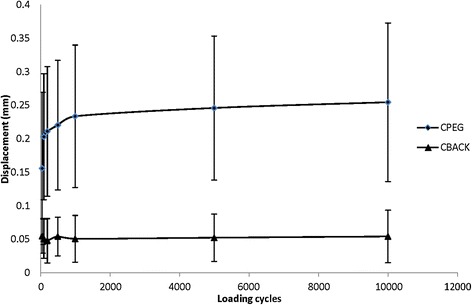
Graph demonstrates initial increase in displacement in CPEG implants with increasing cycles. This gradual increase in displacement plateaus as the number of cycles increase. There is no appreciable difference in initial of final displacement with CBACK components

The CPEG implants had significant and progressive displacement throughout the cyclic testing protocol (Fig. 7). Using a Bonferroni correction for multiple comparisons, the mean difference (0.017 mm) was significant between 100 and 500 cycles (*p* = 0.019), as well as the difference (0.03 mm) between 100 and 1000 cycles (*p* = 0.029). In contrast, there was no significant difference in displacement of the CBACK components throughout the protocol (*p* = 0.45). The measured displacement occurred between the optical trackers fixed to the inferior portion of the glenoid component and the host glenoid bone.

### Micro-CT assessments

In the 16 scapular specimens, there was no significant change in appearance of the polyethylene/cement/glenoid bone interface when comparing the eight zones of interest (*p* = 0.14). Cement mantle thickness ranged from 1.2 to 2.0 mm for all CBACK specimens. Cement mantle fracture was not observed in any specimen and cement mantle defects observed after initial cementation did not progress or change after the loading protocol. Three CBACK specimens had 1-mm radiolucent lines at sites 3, 5, and 8 (anterior position) of the subchondral surface after loading. Specimen #3 demonstrated radiolucent lines in zones 3 and 8. No significant changes were observed at the superior, inferior, or posterior positions. There were no changes to the bone under the cement mantle indicative of bony compression or fracture. There was no appreciable change in polyethylene shape when comparing pre and post micro-CT scans (Table 2).

**Table 2:** Change in appearance of radiolucent lines for each zone

	Zone							
Specimen #	1	2	3	4	5	6	7	8
1 (CPEG)	0	0	0	0	0	0	0	0
2 (CPEG)	0	0	0	0	0	0	0	0
3 (CBACK)	0	0	1	0	0	0	0	1
4 (CBACK)	0	0	0	0	1	0	0	0
5(CPEG)	0	0	0	0	0	0	0	0
6 (CPEG)	0	0	0	0	0	0	0	0
7 (CPEG)	0	0	0	0	0	0	0	0
8 (CBACK)	0	0	0	0	0	0	0	1
9 (CPEG)	0	0	0	0	0	0	0	0
10 (CPEG)	0	0	0	0	0	0	0	0
11 (CBACK)	0	0	0	0	0	0	0	0
12 (CPEG)	0	0	0	0	0	0	0	0
13 (CPEG)	0	0	0	0	0	0	0	0
14 (CBACK)	0	0	0	0	0	0	0	0
15 (CBACK)	0	0	0	0	0	0	0	0
16 (CBACK)	0	0	0	0	0	0	0	1

1 indicates the presence of new radiolucent line and 0 indicates no change

## Discussion

Establishing a cyclic loading protocol and method for determining displacement of polyethylene components in total shoulder arthroplasty can be valuable when evaluating new designs [23–25]. We developed a testing model that is capable of assessing displacement of components dynamically during cyclic loading. Micro-CT scans were useful to confirm that there was no gross abnormality of the cement mantle prior to cyclic testing and at the end of the protocol. The fact that there were no cement mantle fractures was surprising to us, as we theorized that the thin cement mantle would likely fracture during cyclic loading.

The optical tracking during cyclic loading produced several interesting findings related to glenoid component displacement. Implants inserted with the CPEG technique had an initial “setting in” of the component during the first 1000 cycles and thereafter the rate of gradual lift-off diminished but did not cease. This indicates that there was ongoing displacement of the implant relative to the glenoid bone that could represent an early mode of failure with this technique.

Radiostereometric analysis (RSA) has been used to measure in vivo implant displacement following total hip and knee arthroplasty [26]. Two displacement patterns emerge; either the implant achieves solid initial fixation after a brief period of “setting in” or the implant continues to displace. The latter scenario is predictive of catastrophic failure in polyethylene tibial components [27, 28]. A similar conclusion may possibly be drawn here where significant initial movement of the CPEG implant may be predictive of accelerated failure when compared with the CBACK technique that demonstrated no movement.

The observation of implant displacement away from the glenoid bone was not associated with failure and overt loosening in our study as confirmed with the micro-CT data. We are concerned that the initial implant displacement persisted albeit at a diminished rate during extended cyclical loading. It has been shown previously that any tensile force or distraction at a bone cement interface may impact upon long-term implant survival [29]. What we observed could represent a mode of failure whereby synovial fluid accesses and egresses from the space between the implant and host bone.

Many authors have stressed that the initial stability of the implant may be a major determinant for long-term survival [15, 9]. Our results indicated that implant displacement away from the glenoid bone was not observed with the CBACK cementing technique. This may indicate better fixation and potentially improved survivability. The presence of radiolucent lines however in 3 of the 7 CBACK specimens, although not statistically significant, is an interesting observation. Although the loading mechanical properties were not affected in vitro, over time, these radiolucent lines may generate particulate debris that can initiate the cascade leading to osteolysis.

Movement of the inferior portion of the polyethylene away from the glenoid subchondral bone as was observed with limited cementation or the CPEG group may be a suboptimal environment for long-term fixation due to the gradual worsening lift-off and possible fluid egress into the bone cement interface. Although the initial displacement trend decreases after the first 1000 cycles, the implant continues to move relative to the tracker on the bone and this trend may either continue slowly or lead to eventual failure. The initial and sustained stability observed with the CBACK components throughout the loading protocol was superior and warrants further in vivo investigation.

The major limitation of this work was using a loading protocol that represented 5 high load activities per day. This is the equivalent of 150 % body weight 5 times per day for 6 years. The loading protocol may underestimate the actual loads the implant is subjected to during normal day-to-day activities particularly if joint replacements are performed in a younger population. If we assumed double the number of high load activities then our protocol would only represent cyclic loads that the prosthesis is exposed to during a 3-year period. Concerns that specimen degradation may occur during testing precluded prolonging the cyclic loading portion of the testing protocol. Specimen preparation took roughly 12 h in total in addition to the loading protocol. Future study may need to focus on much higher numbers of cycles and perhaps even loading specimens to failure with cycling. An additional limitation with this study and future studies using cyclic loading will be the ongoing accuracy and the potential error of the cyclic loading data with respect to the optical tracking system.

## Conclusion

Total shoulder arthroplasty is an important pain-relieving operation and we must continue to develop implants and optimize implantation techniques that enhance implant survivorship. The lift-off or displacement of the CPEG implants that was observed during the dynamic testing protocol is concerning and may be associated with glenoid loosening. Further in vitro and in vivo testing and analysis are required to determine the long-term survival of current cementing techniques.
